# Supplementary material for: Mutualism breakdown underpins evolutionary rescue in an obligate cross-feeding bacterial consortium
Source: Nat Commun. 2025 Apr 12;16:3482. doi: 10.1038/s41467-025-58742-1 (PMC11992082; doi:10.1038/s41467-025-58742-1)
Supplement: Supplementary file 1 — Supplementary Information [file 41467_2025_58742_MOESM1_ESM.pdf]

Supplementary information for:

Mutualism breakdown underpins evolutionary rescue in an obligate cross-feeding bacterial consortium

Ignacio J. Melero-Jiménez[1,2,3*], Yael Sorokin[1], Ami Merlin[1], Jiawei Li[1], Alejandro Couce[3*†] and Jonathan Friedman[1*†]

[1] *Institute of Environmental Sciences, The Hebrew University of Jerusalem, Rehovot, Israel*

[2] *Departamento de Botánica y Fisiología Vegetal, Universidad de Málaga, Campus de Teatinos s/n, 29071 Málaga, Spain*

[3] *Centro de Biotecnología y Genómica de Plantas (CBGP, UPM-INIA/CSIC), Universidad Politécnica de Madrid (UPM), 28223 Madrid, Spain*

*Corresponding author. Email: imelero@uma.es (I.J.M.J); a.couce@upm.es (A.C.); yonatan.friedman@mail.huji.ac.il (J.F.)

† These authors contributed equally to this work.

The SI includes:
- Supplementary figures 1-11
- Supplementary tables 1-6

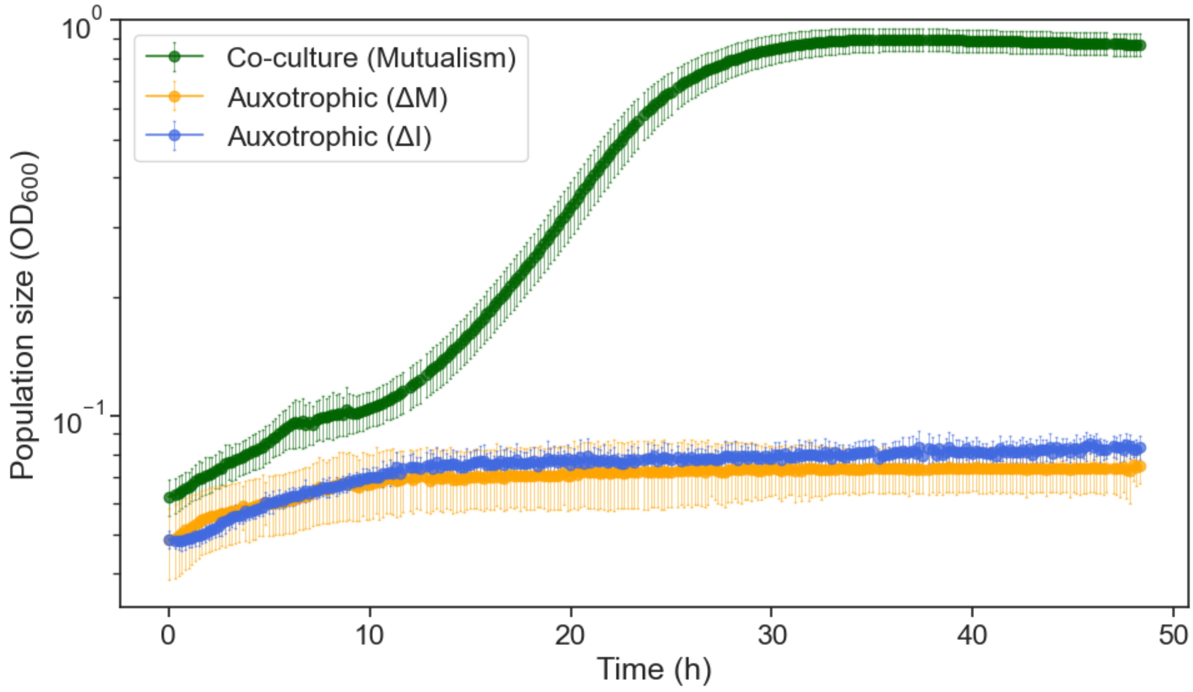

Supplementary Fig. 1: Obligate metabolic cross feeding of auxotrophic strains. The growth curves depict the *E. coli* auxotrophic strains ΔM, ΔI, and their coculture. The data are presented as the mean ± SD (n = 12). Source data are provided as a Source Data file.

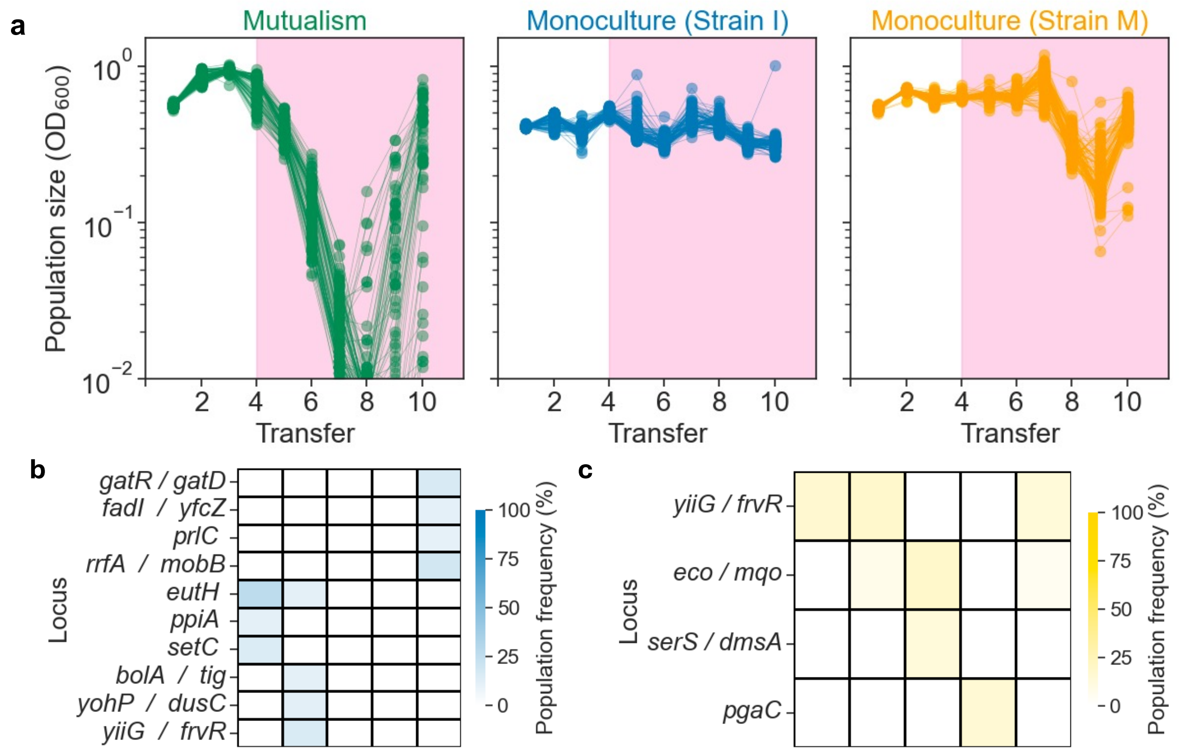

Supplementary Fig. 2: Auxotrophic strains are unaffected by stress when supplemented with amino acids. a Population dynamics of the obligate mutualism consortium (green) and auxotrophic strains I and M (blue and yellow, respectively). The pink background indicates exposure to salinity. The experiment consisted of 96 independent populations for each treatment. The media was supplemented with 100 µM of isoleucine or methionine, respectively. **b, c)** Genes with mutations present in at least two replicate populations or occurring at a frequency > 0.1 in five independent evolutionary replicates from monocultures I (blue) and M (yellow), respectively. Color shades indicate the frequency of each mutation, with darker shades indicating higher mutation frequencies. Specific mutations are detailed in Supplementary Table 5 and 6. Source data are provided as a Source Data file.

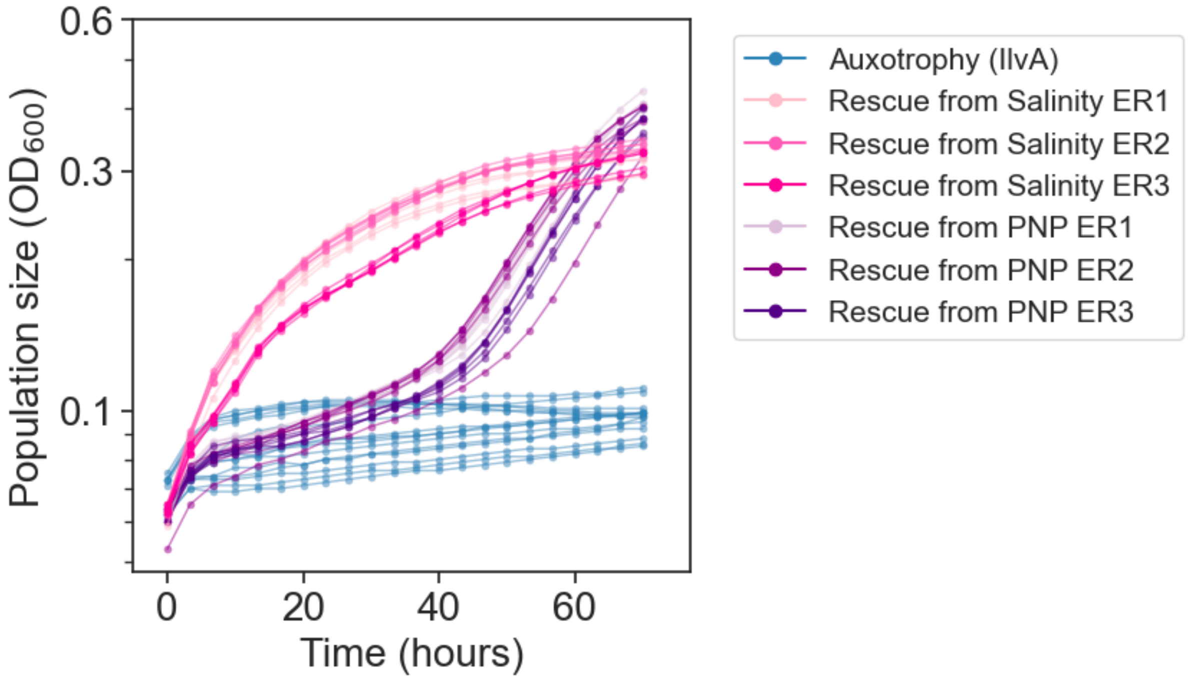

Supplementary Fig. 3: Rescue strains can grow without isoleucine supplied. Growth curves of recovered populations and of the auxotrophic ancestor of the ΔI strain in M9 without isoleucine addition. Different range of colors indicate the treatment from which the populations were isolated (purple from PNP, pink from salinity, and blue is the ancestor ΔI strain). ER denotes an evolutionary replicate. Source data are provided as a Source Data file.

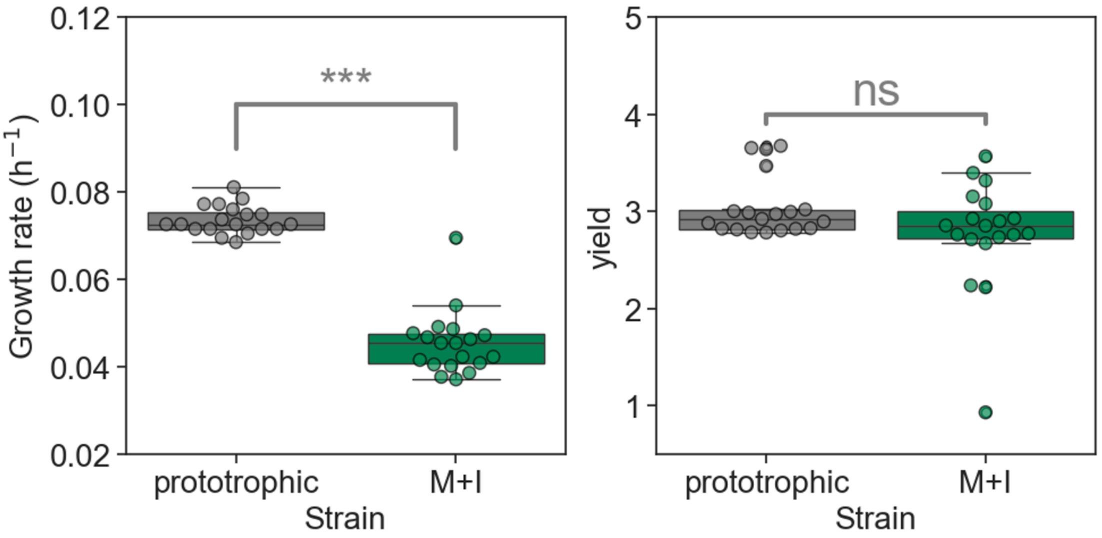

Supplementary Fig. 4: Comparison of Growth rate (h^{-1}) and yield between prototrophic and ΔM+ΔI (mutualism) Strains without stress. Boxplots show growth rate and yield of the prototrophic and ΔM+ΔI strains. Each box plot displays the interquartile range (IQR) of the data, with the horizontal line inside the box indicating the median. The whiskers extend to 1.5 times the IQR, showing the range of the data distribution (n = 19). *P* values (***$p < 0.001$, ns not significant) were determined by a two-sided Mann–Whitney U test. Source data and exact p-values are provided as a Source Data file.

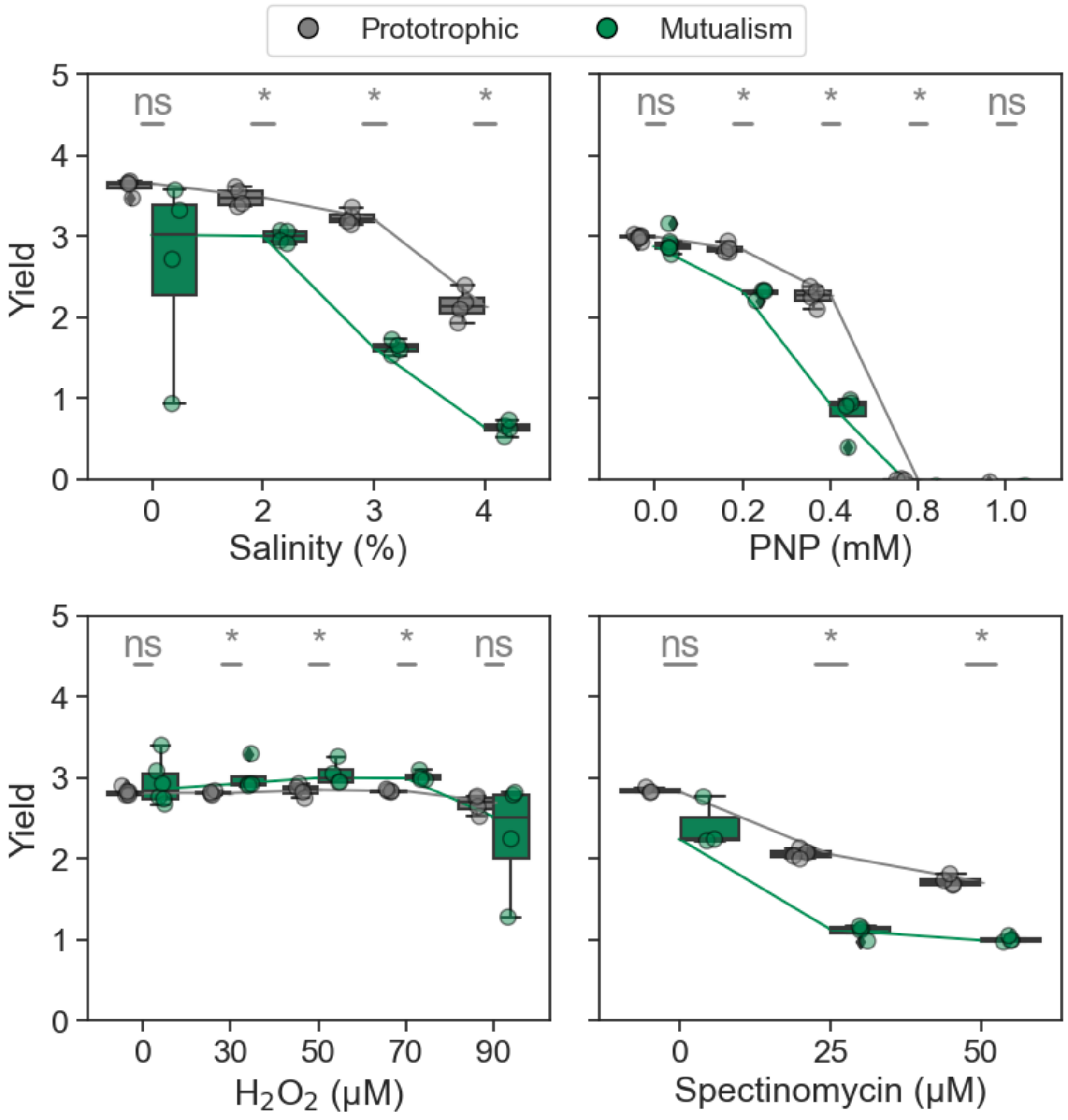

Supplementary Fig. 5: The obligate mutualism is more susceptible to environmental stress than the prototroph. Each panel shows the yield of prototrophic (gray) and mutualistic community (green) under different stressors: salinity (%), p-nitrophenol (PNP, mM), hydrogen peroxide (H_2O_2, μM), and spectinomycin (μM). Each box plot displays the interquartile range (IQR) of the data, with the horizontal line inside the box indicating the median. The whiskers extend to 1.5 times the IQR, representing the range of the data distribution (n = 4 for all boxplots except PNP (0) and H_2O_2 (0), where n = 6, and spectinomycin (0), where n = 3). The lines connecting the boxplots in each panel indicate the median yields for the prototrophic and mutualistic groups across different conditions. P values (*$p < 0.05$, ns not significant) were determined by a two-sided Mann–Whitney U test. Source data and exact p-values are provided as a Source Data file.

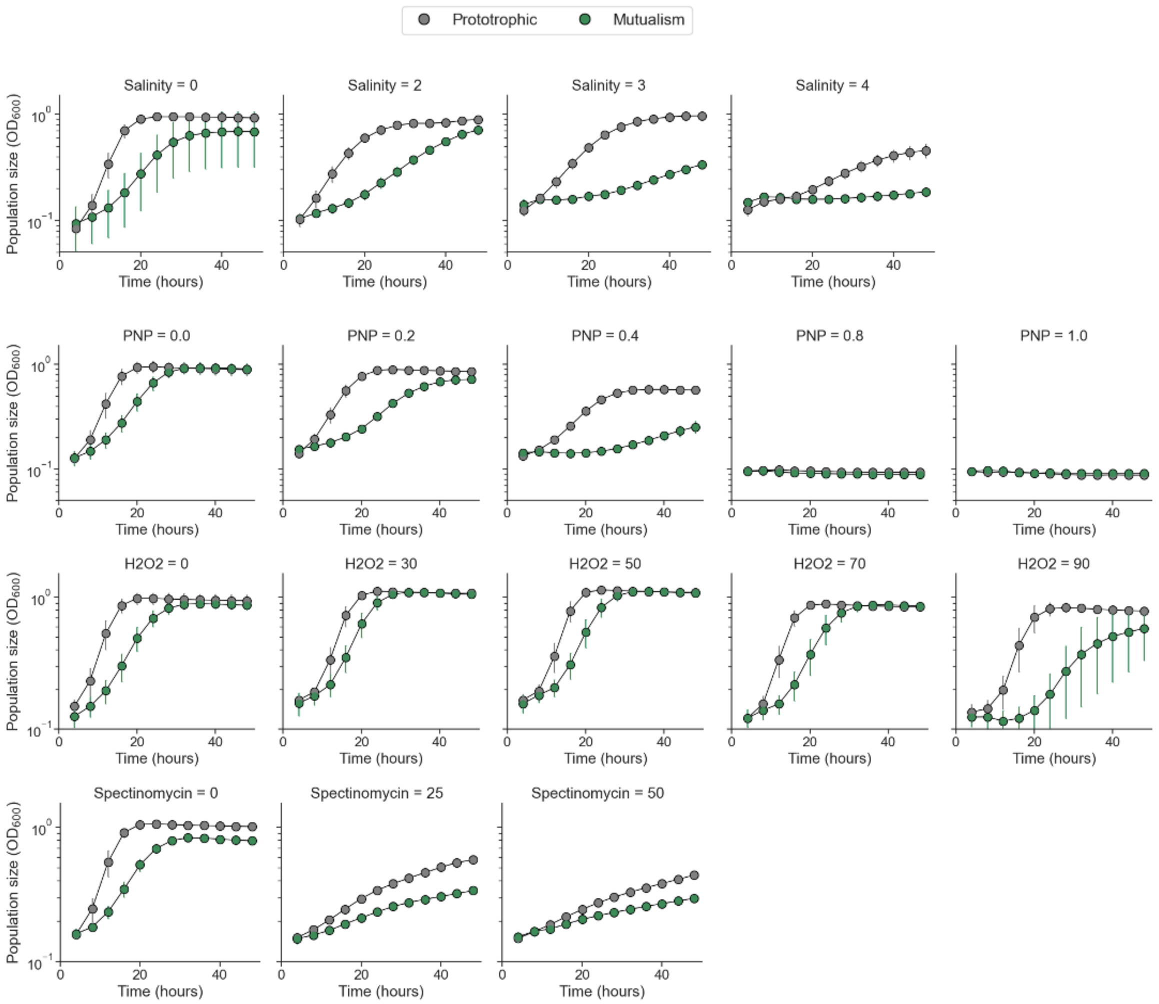

Supplementary Fig. 6: Mutualisms are more sensitive to stress than the prototrophic strain. Growth curves of the prototrophic strain and the mutualism under non-stress and different stressors: salinity (%), p-nitrophenol (PNP, mM), hydrogen peroxide (H_2O_2, μM), and spectinomycin (μM). Data showed the mean ± SD ($n = 4$). Source data are provided as a Source Data file.

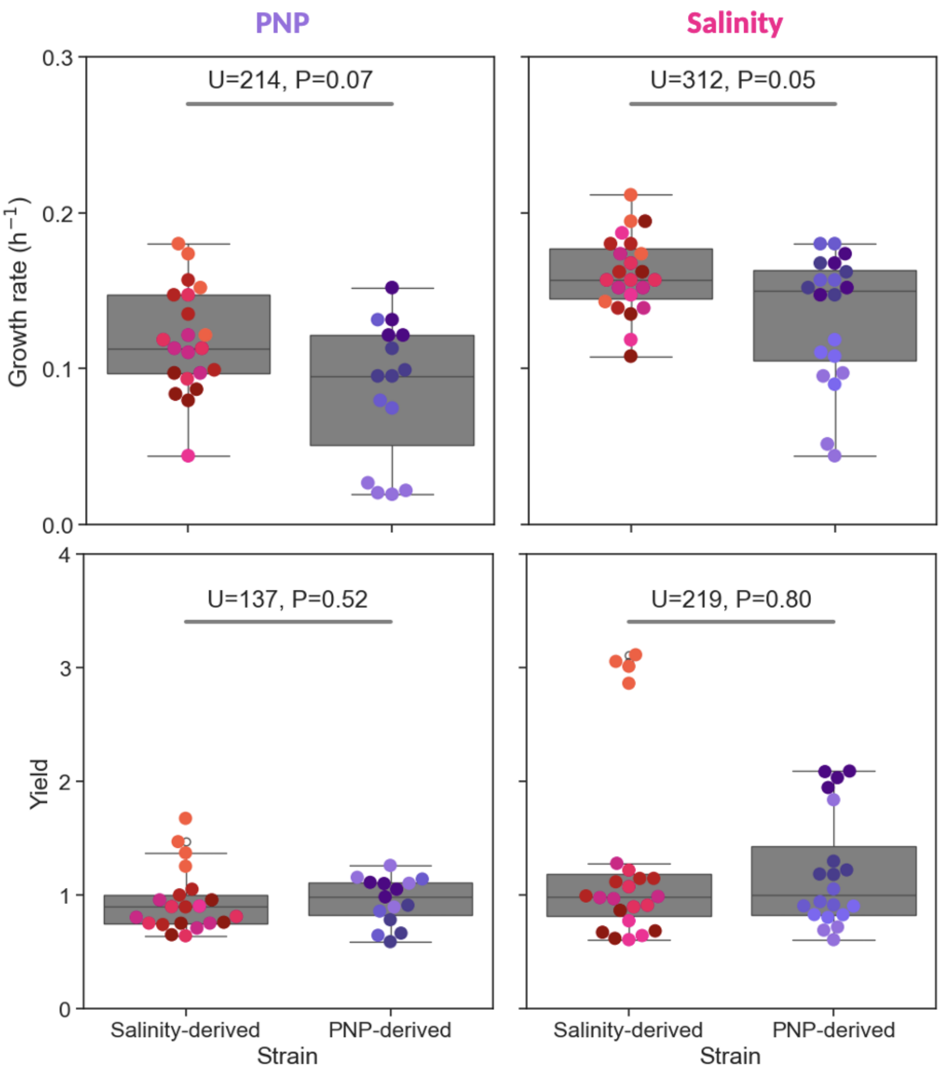

Supplementary Fig. 7: Growth rate and the yield of the rescue strains are similar regardless of their evolutionary history. Each column represents the stress condition under which the isolate-derived strains were grown. Each box plot displays the interquartile range (IQR) of the data, with the horizontal line inside the box indicating the median. The whiskers extend to 1.5 times the IQR, showing the range of the data distribution. Points represent different biological replicates (6 for salinity and 5 for PNP) and technical replicates (3) are indicated with different tones of purple (PNP-derived) or pink (Salinity-derived). The statistical test performed was a two-sided Mann–Whitney U test. Source data are provided as a Source Data file.

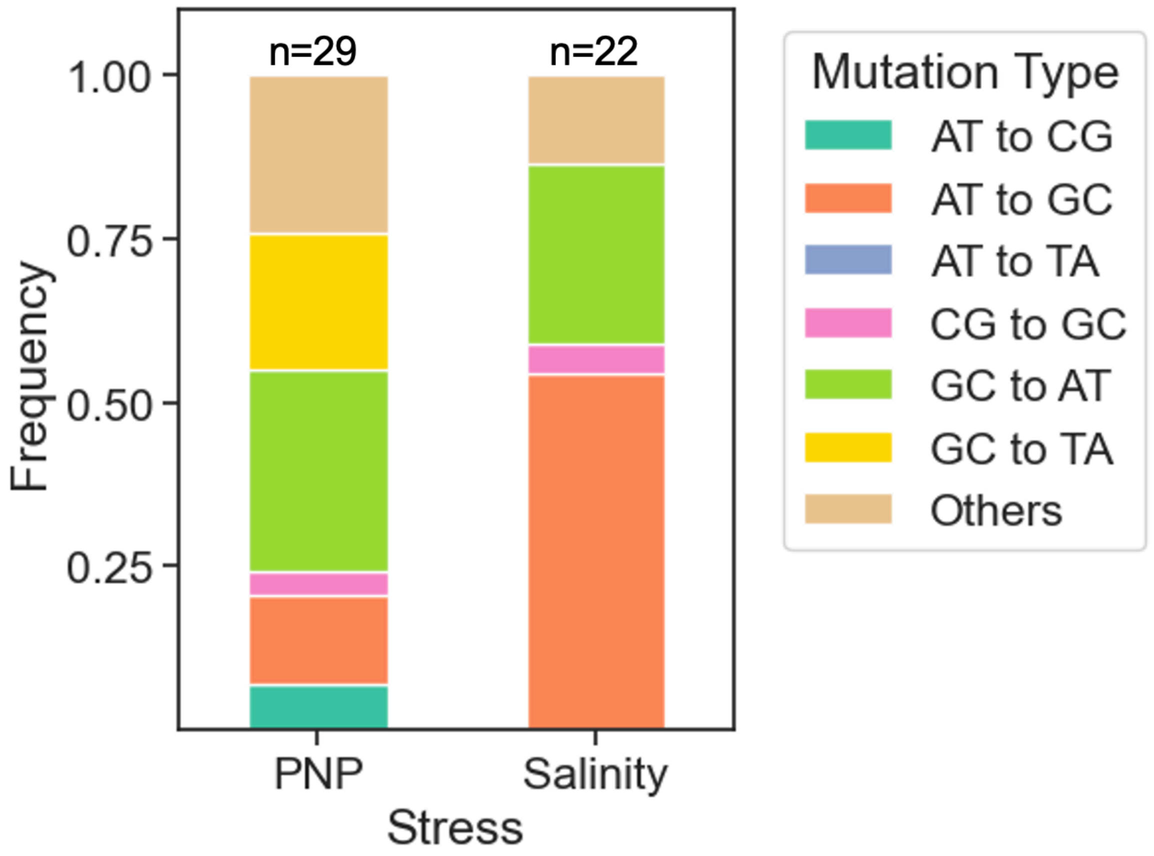

Supplementary Fig. 8: Environmental stresses we used did not introduce significant biases in the overall mutation rates. Base substitution frequencies observed in the genomes of strains evolved under salinity or PNP stress. Colors indicate the six possible substitutions. The specific mutations are listed in Supplementary Table 1 and 2. Source data are provided as a Source Data file.

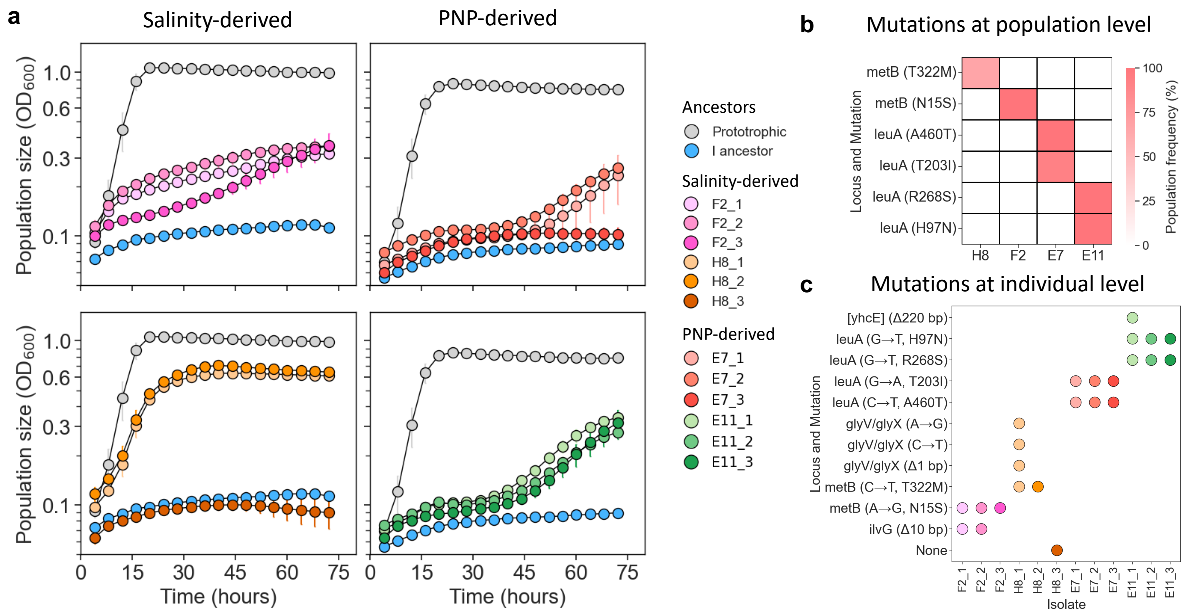

Supplementary Fig. 9: Mutations in metabolic genes can bypass isoleucine auxotrophy and restore growth to varying degrees in the absence of amino acids. a Growth curves of different isolates in media without amino acid supplementation and without stress. In each panel, three different isolates from an independently derived population are shown, together with the prototrophic strain (grey), and the ΔI ancestor (blue). The data are presented as the mean ± SD of 3 replicates per strain. **b** Loci with mutations present in at least two replicate populations or occurring at a frequency > 0.3 and **c)** Loci with mutations present at isolate level. Colors correspond to each isolate. Source data are provided as a Source Data file.

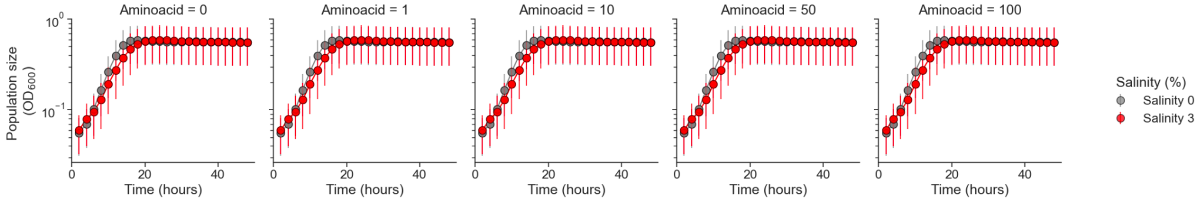

Supplementary Fig. 10: Sensitivity to salinity is not affected by amino acids content for the prototrophic strain. Growth curves of the prototrophic strain without salinity and with 3% salinity at different levels of isoleucine (µM) supplemented to the media. Each panel shows the growth curve at different isoleucine concentration, as indicated by the panel title (gray and red circles indicate 0% and 3% salinity, correspondingly). The data are presented as the mean ± SD (n = 4). Source data are provided as a Source Data file.

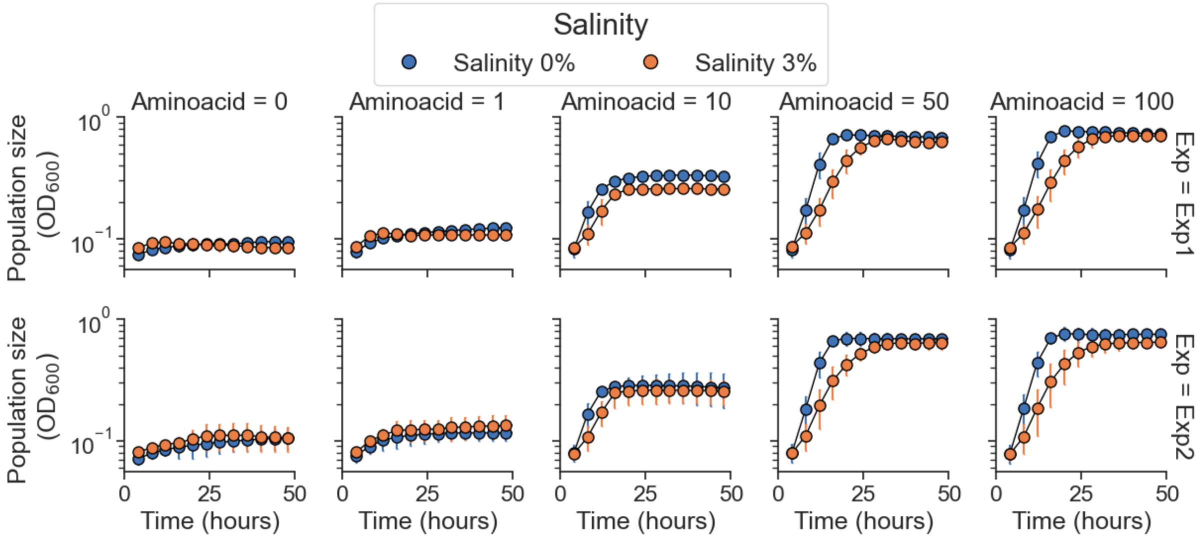

Supplementary Fig. 11: Sensitivity to salinity is not affected by amino acids content for the auxotrophic ΔI strain. Growth curves of the auxotrophic ΔI strain without salinity under non-stress and with 3% salinity (3%) at different levels of isoleucine (μM) supplemented to the media. Each row corresponds to one of two independent experiments. Each panel depicts the growth curve at different isoleucine concentration (blue circles for 0% and orange circles for 3% salinity). The data are presented as the mean ± SD (n = 4). Source data are provided as a Source Data file.

Supplementary Table 1. List of primers used in this study.

Gene	Primer sequence
ilvA_vf	GTGAACGTCAGGTCTCCTTTG
ilvA_vr	TATGCGCCGCGCAGC
metA_vf	TCACCTTCAACATGCAGGC
metA_vr	ATATCGACCTGCAAAGGTGAGT

Supplementary Table 2. List of mutations found in the ΔI strain derived from the evolutionary rescue experiment with salinity.

Locus	mutation	annotation	population	description
cysE ←	C→T	A28T (GCC→ACC)	3	serine acetyltransferase
	G→A	R125C (CGT→TGT)	5	
	A→G	Y31H (TAC→CAC)	7	
	IS*1* (+) +9 bp	coding (780-788/822 nt)	11	
ilvG →	$(AAATC)_{1\to2}$	pseudogene (979/984 nt)	13	pseudogene, acetohydroxy acid synthase II; acetolactate synthase II, large subunit, cryptic, interrupted
	Δ1 bp	|pseudogene (1/663 nt)	14	
metB	A→G	N15S (AAT→AGT)	2	cystathionine gamma-synthase, PLP-dependent
	A→G	N15S (AAT→AGT)	6	
	C→T	T322M (ACG→ATG)	8	
	C→T	T322M (ACG→ATG)	9	
	$(TTACTC)_{1\to2}$	coding (140/1161 nt)	10	
	A→G	N15S (AAT→AGT)	12	
	A→G	N15S (AAT→AGT)	13	
	A→G	N15S (AAT→AGT)	14	
	A→G	N15S (AAT→AGT)	15	
metC →	A→C	D185A (GAC→GCC)	4	cystathionine beta-lyase, PLP-dependent
ybjE ← / ← *aqpZ*	T→C	intergenic (-369/+126)	1	putative transporter/aquaporin Z
	C→G	intergenic (-341/+154)	7	
	T→C	intergenic (-369/+126)	11	
	T→C	intergenic (-369/+126)	13	
	T→C	intergenic (-369/+126)	14	
yehU	G→A	L375F (CTT→TTT)	8	putative sensory kinase in two-component system with YehT, inner membrane protein
	G→A	T325I (ACC→ATC)	10	
yfaL ←	Δ18 bp	coding (2791-2808/3753 nt)	4	adhesin

Supplementary Table 3. List of mutations found in the ΔI strain derived from the evolutionary rescue experiment with PNP.

Locus	mutation	annotation	population	description
betT → / → *yahA*	T→C	intergenic (+386/-489)	12	2-isopropylmalate synthase
hfq →	G→T	K31N (AAG→AAT)	5	global sRNA chaperone; HF-I, host factor for RNA phage Q beta replication
ilvB ←	+A	coding (623/1689 nt)	6	acetolactate synthase I, large subunit
	G→T	S8* (TCG→TAG)	14	
leuA ←	C→T	A460T (GCG→ACG)	1	2-isopropylmalate synthase
	G→A	R268C (CGC→TGC)	3	
	G→A	A453V (GCC→GTC)	4	
	C→T	A460T (GCG→ACG)	5	
	C→T	G462S (GGT→AGT)	5	
	C→T	G427D (GGT→GAT)	6	
	A→G	S400P (TCT→CCT)	7	
	G→T	R268S (CGC→AGC)	8	
	G→T	H97N (CAT→AAT)	8	
	C→T	A460T (GCG→ACG)	9	
	G→A	T203I (ACC→ATC)	9	
	C→A	G462V (GGT→GTT)	10	
	A→C	S488A (TCT→GCT)	11	
	Δ12 bp	coding (1356-1367/1572 nt	12	
	C→A	G462V (GGT→GTT)	13	
	C→T	A460T (GCG→ACG)	14	
	A→C	L461R (CTG→CGG)	15	
lrp →	C→T	P87L (CCG→CTG)	10	DNA-binding transcriptional dual regulator, leucine-binding
	G→T	R137L (CGT→CTT)	11	
	C→T	P87L (CCG→CTG)	13	
rpoS ←	Δ1 bp	coding (10/993 nt)	1	RNA polymerase, sigma S (sigma 38) factor
ybjE ← / ← *aqpZ*	T→C	intergenic (-369/+126)	3	putative transporter/aquaporin Z
	T→C	intergenic (-369/+126)	12	
	C→G	intergenic (-341/+154)	10	

Supplementary Table 4. Colony counts and optical density (OD_{600}) of Strain M and I engaged in mutualism under non-stress. The growth experiment was performed in 96 well plates (200 ul). The initial OD_{600} of cocultures (ΔM and ΔI) was 0.025 of each strain. The OD_{600} was measured in Synergy microplate reader (BioTek) and was recorded using Gen5 v3.09 software (BioTek). Plates were incubated at 30 °C with a 1 °C every gradient to avoid condensation on the lid and were shaken at 250 rpm. OD_{600} was measured 5 min for 48h. Then, cultures were diluted to a factor of 10^6 and 100 µL were plated in agar plates of M9 agar plates with methionine or isoleucine. Plates were incubated at 30 °C for 48h and colonies were counted manually. We previously checked that ΔM and ΔI cannot growth in plates without methionine or isoleucine respectively. Colonies in methionine plates correspond with the strain ΔM while colonies in isoleucine plates correspond with the strain ΔI.

Strain	Final OD_{600}	Colonies on Methionine Plate	Colonies on Isoleucine Plate
M+I	0.75	13	21
M+I	0.78	6	17
M+I	0.79	11	26
M+I	0.73	12	27
M+I	0.74	21	38

Supplementary Table 5. List of mutations found in the ΔI strain after being grown in monoculture with isoleucine supplementation and exposed to salinity.

Locus	mutation	annotation	population	description
gatR ← / ← gatD	T→C	Intergenic (-88/+18)	C5	Pseudogene, repressor for gat operon; split galactitol utilization operon repressor, fragment 2; interrupted by IS3/galactitol-1-phosphate dehydrogenase, Zn-dependent and NAD(P)-binding
fadI ← / ← yfcZ	C→A	Intergenic (-108/+73)	C5	Beta-ketoacyl-CoA thiolase, anaerobic, subunit/conserved protein, UPF0381 family
prlC ←	C→A	C446F (TGT→TTT)	C5	Oligopeptidase A
rrfA → / ← mobB	T→C	Intergenic (+173/+97)	C5	5S ribosomal RNA of rrnA operon/molybdopterin-guanine dinucleotide biosynthesis protein B
eutH ←	G→T	P393Q (CCA→CAA)	F2	Ethanolamine transporter
ppiA ←	A→C	L44R (CTG→CGG)	F2	Peptidyl-prolyl cis-trans isomerase A (rotamase A)
setC →	C→G	Y104* (TAC→TAG)	F2	Putative arabinose efflux transporter
bolA → / → tig	G→T	Intergenic (+93/-251)	D7	Stationary-phase morphogene, transcriptional repressor for mreB; also regulator for dacA, dacC, and ampC/peptidyl-prolyl cis/trans isomerase (trigger factor)
yohP → / ← dusC	C→T	Intergenic (+118/+255)	D7	Hypothetical protein/tRNA-dihydrouridine synthase C
yiiG → / ← frvR	T→G	Intergenic (+30/+20)	D7	Conserved lipoprotein/putative frv operon regulator; contains a PTS EIIA domain

Supplementary Table 6. List of mutations found in the ΔM strain after being grown in monoculture with methionine supplementation and exposed to salinity.

Locus	mutation	annotation	population	description
yiiG → / ← *frvR*	A→C	Intergenic (+38/+12)	G4	Conserved lipoprotein/putative frv operon regulator; contains a PTS EIIA domain
	T→G	Intergenic (+30/+20)	H6	
	T→G	Intergenic (+30/+20)	E7	
eco → / ← *mqo*	A→G	Intergenic (+295/+420)	B10	Ecotin, a serine protease inhibitor/malate dehydrogenase, FAD/NAD(P)-binding domain
	A→G	Intergenic (+620/+95)	E7	
serS → / → *dmsA*	T→G	Intergenic (+40/-199)	B10	Seryl-tRNA synthetase, also charges selenocysteinyl-tRNA with serine/dimethyl sulfoxide reductase, anaerobic, subunit A
pgaC ←	T→G	I77L (ATC→CTC)	D12	Biofilm PGA synthase PgaCD, catalytic subunit;poly-beta-1,6-N-acetyl-D-glucosamine synthase; c-di-GMP-stimulated activity and dimerization
