# Supplementary material for: Mutualism breakdown underpins evolutionary rescue in an obligate cross-feeding bacterial consortium
Source: Nat Commun. 2025 Apr 12;16:3482. doi: 10.1038/s41467-025-58742-1 (PMC11992082; doi:10.1038/s41467-025-58742-1)
Supplement: Supplementary file 2 — Reporting Summary [file 41467_2025_58742_MOESM2_ESM.pdf]

Corresponding author(s): Ignacio José Melero Jiménez, Alejandro Couce and Jonathan Fridman

Last updated by author(s): Mar 10, 2025

Reporting Summary

Nature Portfolio wishes to improve the reproducibility of the work that we publish. This form provides structure for consistency and transparency in reporting. For further information on Nature Portfolio policies, see our Editorial Policies and the Editorial Policy Checklist.

Statistics

For all statistical analyses, confirm that the following items are present in the figure legend, table legend, main text, or Methods section.

n/a	Confirmed	
☐	☒	The exact sample size (*n*) for each experimental group/condition, given as a discrete number and unit of measurement
☐	☒	A statement on whether measurements were taken from distinct samples or whether the same sample was measured repeatedly
☐	☒	The statistical test(s) used AND whether they are one- or two-sided<br>*Only common tests should be described solely by name; describe more complex techniques in the Methods section.*
☐	☒	A description of all covariates tested
☐	☒	A description of any assumptions or corrections, such as tests of normality and adjustment for multiple comparisons
☐	☒	A full description of the statistical parameters including central tendency (e.g. means) or other basic estimates (e.g. regression coefficient) AND variation (e.g. standard deviation) or associated estimates of uncertainty (e.g. confidence intervals)
☐	☒	For null hypothesis testing, the test statistic (e.g. *F*, *t*, *r*) with confidence intervals, effect sizes, degrees of freedom and *P* value noted<br>*Give P values as exact values whenever suitable.*
☒	☐	For Bayesian analysis, information on the choice of priors and Markov chain Monte Carlo settings
☒	☐	For hierarchical and complex designs, identification of the appropriate level for tests and full reporting of outcomes
☐	☒	Estimates of effect sizes (e.g. Cohen's *d*, Pearson's *r*), indicating how they were calculated

Our web collection on statistics for biologists contains articles on many of the points above.

Software and code

Policy information about availability of computer code

Data collection	Optical density measurements were done using BioTek intruments Gen 5 v3.09 software. We extracted genomic DNA using the Norgen Biotek Corp Bacterial Genomic DNA Isolation Kit (Cat No. 17900) and sent it to the sequencing facility at SEQCENTER (PA, USA, https://www.seqcenter.com/).
Data analysis	The Python 3.11.9 code used for analyzing, and creating figures is open-source and available on GitHub (https://github.com/ignamel/ER_mutualism.git) and stored at https://doi.org/10.5281/zenodo.1499267063

For manuscripts utilizing custom algorithms or software that are central to the research but not yet described in published literature, software must be made available to editors and reviewers. We strongly encourage code deposition in a community repository (e.g. GitHub). See the Nature Portfolio guidelines for submitting code & software for further information.

Data

Policy information about availability of data

All manuscripts must include a data availability statement. This statement should provide the following information, where applicable:

- Accession codes, unique identifiers, or web links for publicly available datasets
- A description of any restrictions on data availability
- For clinical datasets or third party data, please ensure that the statement adheres to our policy

The full dataset used in this study is available on GitHub at https://github.com/ignamel/ER_mutualism.git and on Zenodo at https://doi.org/10.5281/

zenodo.1499267063. Source data are provided as a Source Data file. All raw sequencing data is available on the US National Center for Biotechnology Information (NCBI) Sequence Read Archive (SRA) under BioProject PRJNA1143481 (http://www.ncbi.nlm.nih.gov/bioproject/1143481).

Research involving human participants, their data, or biological material

Policy information about studies with human participants or human data. See also policy information about sex, gender (identity/presentation), and sexual orientation and race, ethnicity and racism.

Reporting on sex and gender	NA
Reporting on race, ethnicity, or other socially relevant groupings	NA
Population characteristics	NA
Recruitment	NA
Ethics oversight	NA

Note that full information on the approval of the study protocol must also be provided in the manuscript.

Field-specific reporting

Please select the one below that is the best fit for your research. If you are not sure, read the appropriate sections before making your selection.

☐ Life sciences ☐ Behavioural & social sciences ☒ Ecological, evolutionary & environmental sciences

For a reference copy of the document with all sections, see nature.com/documents/nr-reporting-summary-flat.pdf

Ecological, evolutionary & environmental sciences study design

All studies must disclose on these points even when the disclosure is negative.

Study description	This study included two bacterial genotypes derived from the bacterial strain Escherichia coli EcNR1. Each strain was auxotrophic for one of two different amino acids (methionine and isoleucine) and derived from a previous study (Mee et al., 2014, PNAS). Here, we aimed to explore how interactions between bacteria can affect the possibility of evolutionary rescue. We perform an evolution experiment where monocultures of the prototrophic strain and cocultures of the auxotrophic strains were exposed to two different stresses: salinity and PNP. The growth of all cultures was tracked by quantifying their population density (OD600 nm) while propagating them to fresh medium every 48 hours. We also checked the population composition and detected that evolutionary rescue occurred due to the breakdown of mutualism. Finally, we explored the mutations that allow auxotrophic E. coli to bypass the function of the knockout gene.
Research sample	Escherichia coli used in this study were based on the EcNR1 E. coli derivative of MG1655. We used the auxotrophic strains ΔmetA, ΔilvA, and the WT. The amino acid auxotrophs were generated by Red-recombineering with a chloramphenicol resistance cassette. Information and strains were obtained from Mee et al. (2014, PNAS). We chose these strains because they have been previously demonstrated to reciprocally exchange amino acids and engage in obligate mutualism. These strains were then used to inoculate 46 replicates for each of the two cultures: (1) monoculture of prototrophic and (2) coculture of ΔI and ΔM. Cultures were grown in a volume of 800 µl in 96-well plates at 30°C and were shaken at 900rpm for 48 hours. Forty µl of each culture were transferred every 48 hours into 760 µl of fresh medium. During the first three growth cycles, no stress was applied to allow populations to reach an equilibium. At the fourth transfer, each test culture was split into three different environments: control (no stress), salinity (3% NaCl), and PNP (0.4 mM). Growth of all cultures was tracked by quantifying their population density (OD600) while propagating them to fresh medium with the specific stress. At the end of the experiment we extract DNA and we sequencing from the evolve populations.
Sampling strategy	An experiment was conducted with 46 replicates for each consortium-treatment combination. In our study, growth was consistently measured as a continuous variable at a specified time point (i.e., 48 hours) post-inoculation. This method allowed us to quantitatively compare the evolutionary paths of various samples.
Data collection	Optical density measurements were done using BioTek intruments Gen 5 v3.09 software. We extracted genomic DNA using the Norgen Biotek Corp Bacterial Genomic DNA Isolation Kit (Cat No. 17900) and sent it to the sequencing facility at SEQCENTER (PA, USA, https://www.seqcenter.com/).
Timing and spatial scale	The evolution experiment lasted for 40 days, and growth was quantified every 48 h, starting with the first inoculation t = 0 h. Evolutionary experiments were performed in 96 deep-well plates (maximal volume: 1ml, Thermo Scientific Nunc).
Data exclusions	Contaminated samples were excluded from the analysis. Negative values were excluded from the analysis of rescue transfer, maximum recovery rate, and final population size because they were irrelevant for the study.
Reproducibility	Each consortium-treatment combination was independently replicated 46-times.

Randomization	No grouping was done in this experiment.
Blinding	As no grouping was done during this study, blinding in the data collection and in the data analysis is not relevant.

Did the study involve field work? ☐ Yes ☒ No

Reporting for specific materials, systems and methods

We require information from authors about some types of materials, experimental systems and methods used in many studies. Here, indicate whether each material, system or method listed is relevant to your study. If you are not sure if a list item applies to your research, read the appropriate section before selecting a response.

Materials & experimental systems

n/a	Involved in the study
☒	☐ Antibodies
☒	☐ Eukaryotic cell lines
☒	☐ Palaeontology and archaeology
☒	☐ Animals and other organisms
☒	☐ Clinical data
☒	☐ Dual use research of concern
☒	☐ Plants

Methods

n/a	Involved in the study
☒	☐ ChIP-seq
☒	☐ Flow cytometry
☒	☐ MRI-based neuroimaging

Plants

Seed stocks	NA
Novel plant genotypes	NA
Authentication	NA
